# A rare case report of uterine carcinosarcoma with bilateral ovarian Brenner tumors

**DOI:** 10.3389/fonc.2025.1612716

**Published:** 2025-08-27

**Authors:** Xinyao Wan, Fangfang Bi, Bing Xin, Chong Qiao

**Affiliations:** Department of Obstetrics and Gynecology, Shengjing Hospital of China Medical University, Shenyang, China

**Keywords:** uterine carcinosarcoma, Brenner tumor, case report, immunohistochemistry, multidisciplinary care

## Abstract

**Background:**

Uterine carcinosarcoma is a rare, highly aggressive malignancy characterized by both carcinomatous and sarcomatous components. Brenner tumors of the ovary are uncommon epithelial neoplasms, usually benign but occasionally coexisting with other pathologies. The co-occurrence of these two entities is extremely rare and poses diagnostic and therapeutic challenges.

**Methods:**

We report a case of a 58-year-old female presenting with scant yellowish vaginal discharge. Imaging studies revealed an intrauterine mass. Histopathological analysis of curettage specimens confirmed endometrial malignancy. The patient underwent radical surgical resection followed by histopathological and immunohistochemical analysis.

**Results:**

Histopathology confirmed uterine carcinosarcoma comprising high-grade endometrial adenocarcinoma and pleomorphic sarcoma with chondrosarcoma differentiation. Bilateral ovarian Brenner tumors were also identified. Given the aggressive nature of carcinosarcoma, the patient was referred for adjuvant therapy.

**Conclusion:**

This case highlights the importance of prompt pathological evaluation in atypical gynecologic presentations. Early diagnosis through histopathology and immunohistochemistry is crucial for managing rare and aggressive tumors such as uterine carcinosarcoma, particularly when coexisting with other uncommon neoplasms like Brenner tumors. Multidisciplinary care and individualized treatment plans are essential for optimizing outcomes.

## 1 Introduction

Uterine carcinosarcoma (UCS), also known as malignant mixed Mullerian tumors (MMMTs), is a rare, highly aggressive biphasic malignancy that includes epithelial carcinoma and stromal sarcoma components. Uterine carcinosarcoma is a biphasic tumor with both carcinomatous and sarcomatous components, including low grade and high grade (1).The tumors included endometrioid carcinoma, serous carcinoma, clear cell carcinoma, squamous cell carcinoma and undifferentiated carcinoma. The sarcoma components included homologous sarcoma, leiomyosarcoma, endometrial stromal sarcoma, undifferentiated sarcoma and fibrosarcoma, and heterogenous sarcoma included rhabdomyosarcoma, osteosarcoma, chondrosarcoma and liposarcoma. Although UCS represents only 2–5% of uterine cancers, it exhibits marked aggressiveness: Even in early-stage disease, 5-year survival rates are 50–60%, and it accounts for approximately 15% of uterine cancer-related deaths (1–3). Ovarian Brenner tumors account for 5% of all ovarian tumors. They usually arise from metaplastic transformation of the ovarian surface epithelium and can occur at any age. They are usually found incidentally in perimenopausal women aged 50 to 60 years. Followed by abdominal pain and postmenopausal vaginal bleeding, other symptoms included nausea, poor vomiting, and constipation, while some patients had no obvious clinical symptoms (4).This report presents a case of a patient with concurrent uterine carcinosarcoma and bilateral ovarian Brenner tumors, offering insight into their potential origin and advancing our understanding of their pathophysiology.

## 2 Case report

### 2.1 Clinical presentation

A 58-year-old postmenopausal woman was admitted to Shengjing Hospital of China Medical University in February 2024, presenting with complaints of irregular vaginal bleeding. She had been menopausal for 7 years, with a history of three induced abortions, one natural delivery, and a 30-year smoking history (10 cigarettes per day). Transvaginal 3D ultrasound revealed endometrial thickening (1.6 cm) with heterogeneous echoes, prompting further investigation. Initially, the patient declined treatment but returned in October 2024 with complaints of vaginal bleeding and yellowish discharge persisting for the past three months. Upon admission, physical examination revealed mild abdominal distension, with normal lung and cardiac findings and no signs of acute distress. A gynecological examination noted a retroverted uterus, which was slightly enlarged and firm. Laboratory tests showed an elevated carbohydrate antigen 125 (CA125) level of 40.4 U/ml(modestly elevated), suggesting a potential malignancy. HPV typing and cervical histology results were negative.

### 2.2 Imaging and diagnostic workup

**Figure 1:**
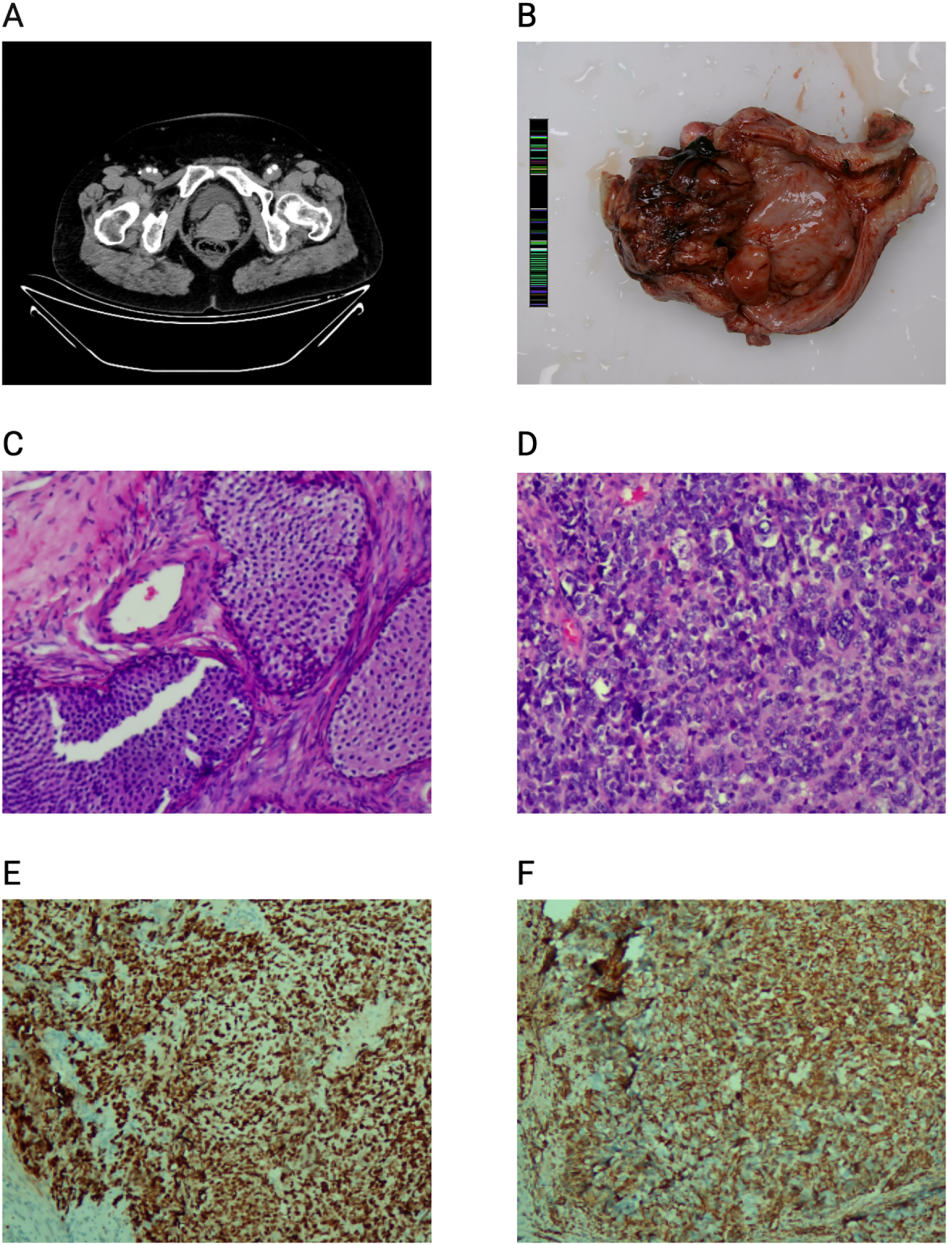
Benign ovarian Brenner tumor coexisting with uterine carcinosarcoma: radiologic, gross, and histologic findings. **(A)** Pelvic CT scan reveals a well-defined, calcified mass in the right adnexal region. **(B)** Gross pathology of the excised ovarian mass measuring 6 cm × 5 cm × 5 cm, showing solid and cystic components with focal hemorrhage. **(C)** Histopathological section of the ovary demonstrates nests of transitional-type epithelial cells embedded in a dense fibrous stroma, consistent with benign Brenner tumor (H&E stain). **(D)** Histological section of the endometrium shows atypical glandular structures with marked nuclear pleomorphism and mitotic activity, consistent with endometrial carcinoma (H&E stain). **(E)** p53 immunohistochemistry, which shows that tumor cells exhibit diffuse strong positive staining for p53, suggesting abnormal p53 protein expression. **(F)** vimentin immunohistochemistry. Tumor cells show positive staining for vimentin, indicating that they have mesenchymal differentiation characteristics. This staining result is helpful for the differential diagnosis and understanding of tumor biological behavior.

Imaging studies, including 3D transvaginal ultrasound and contrast-enhanced CT scans, revealed a markedly thickened endometrium and a possible uterine mass measuring 5.2×5.0 cm (**Figure 1A**). No enlarged pelvic lymph nodes were observed, but small retroperitoneal nodules were noted. A diagnostic curettage performed on October 29, 2024, confirmed malignancy in the endometrial tissue. The patient then underwent a total hysterectomy with bilateral salpingo-oophorectomy, combined with pelvic and para-aortic lymphadenectomy. The postoperative diagnosis indicates a malignant uterine tumor. The surgical procedure involved a midline abdominal incision extending approximately 30 cm. Intraoperatively, the peritoneum appeared smooth with no ascitic fluid. The uterus measured 7.0x6.0x5.0 cm with a thickened myometrium and smooth serosal surface. The right ovary was about 2.0×1.0cm, appearing firm with no visible abnormalities, while the left ovary was about 3.0×2.0 cm, similarly firm and without notable abnormalities. The patient underwent total hysterectomy with bilateral salpingo-oophorectomy, omentectomy, and pelvic and para-aortic lymphadenectomy. Intraoperative blood loss was 100 mL, with no fluid output through suction. Pathological examination confirmed the diagnosis of uterine carcinosarcoma, along with bilateral ovarian Brenner tumors (**Figures 1C–F**).

### 2.3 Gross and microscopic findings

Gross examination of the uterus revealed a protruding mass in the right uterine horn, measuring 6.0×5.0×5.0 cm, with a yellowish-white cut surface (**Figure 1B**). Microscopic examination showed a mixed distribution of malignant epithelial and mesenchymal components, with a predominance of sarcomatous tissue. The malignant mesenchymal component exhibited pleomorphic, atypical nuclei, frequent mitoses, and areas of chondroid matrix formation. The malignant epithelial component contained glandular structures with atypical cells. Brenner tumors were identified in both ovaries, characterized by cell nests within fibromatous stroma, with typical nuclear features, including pale cytoplasm and nuclear grooves. Immunohistochemical staining revealed p53 (approximately 100%+, showing a mutant-type expression); Vimentin (+); p16 (diffusely +); ER (approximately 20%+); PR (approximately 10-20%+); Her-2 (1+); SMARCA4 (+); CK (partially+). MLH1 (+), PMS2 (+), MSH2 (+), MSH6 (+), with no detected loss of mismatch repair (MMR) protein expression in this case (**Table 1**).

**Table 1:** Summary of immunohistochemical (IC) markers and results.

IHC Marker	Result (+/-)	Percent positivity (%)
p53	+	100%
ER (Estrogen Receptor)	+	20%
PR (Progesterone Receptor)	+	10-20%
He 2	+	
Vimentin	+	
P16	+	
MLH1 (MMR protein)	+	
MSH2 (MMR protein)	+	
MSH6 (MMR protein)	+	
PMS2 (MMR protein)	+	

### 2.4 Treatment and follow-up

The patient recovered well postoperatively, without any significant complications. Despite recommendations for adjuvant chemotherapy and radiotherapy, both the patient and her family declined further treatment. Regular follow-up has shown no signs of recurrence at the time of writing.

## 3 Discussion

A systematic search of PubMed, Embase, and Web of Science using keywords related to UCS, ovarian Brenner tumors, and their co-occurrence revealed no documented cases of their simultaneous occurrence. Key case series and literature reviews on ovarian Brenner tumors—including Lou et al.’s analysis of 20 cases and broader syntheses—report associations primarily with endometrial carcinoma, not UCS (5). Similarly, studies examining the pathogenesis, clinical features, and management of UCS have not reported any associations with ovarian Brenner tumors. To our knowledge, this is the first reported case of concurrent UCS and bilateral ovarian Brenner tumors.

UCS is rare malignancy that most commonly affects postmenopausal women aged 60~80 years (6–8).High-risk factors include postmenopausal status, exogenous estrogen exposure, tamoxifen use, nulliparity, and obesity. Clinically, it typically presents with abnormal vaginal bleeding, leukorrhea, uterine enlargement, or abdominal pain (7, 8).While serological and imaging studies effectively detect most malignant uterine tumors, UCS often evades early diagnosis due to nonspecific symptoms that overlap with other uterine malignancies, resulting in advanced-stage presentation in most patients (9).

Treatment of UCS remains challenging owing to its aggressive biology and histopathological complexity. Surgery is the cornerstone of management; for eligible patients, recommended procedures include total hysterectomy, bilateral salpino-oophorectomy, pelvic and/or para-aortic lymphadenectomy, peritoneal lavage with cytological examination, random peritoneal biopsies, and omentectomy. Current evidence indicates that UCS requires more aggressive management than endometrial cancer (10).Since 2017, NCCN guidelines have recommended adjuvant chemotherapy with or without radiotherapy (10, 11).A retrospective SEER database analysis found only a limited survival benefit with radiotherapy in patients with node-negative UCS (HR 0.85, 95%CI 0.70-1.03) (12).Although radiotherapy alone reduces the risk of local recurrence, it does not improve overall survival (OS). A prospective randomized trial demonstrated efficacy of carboplatin/paclitaxel (PC) for stage III-IV disease, with a median OS of 37 months, and a large National Cancer Database (NCDB) study showed improved OS with adjuvant chemoradiotherapy versus observation (HR 0.55, 95%CI 0.46-0.66) (13, 14).Further randomized controlled trials to define the most effective therapeutic strategies are essential (15).Additionally, given the high rates of local recurrence and distant metastasis, targeted therapy and immunotherapy represent active areas of research, with a focus on elucidating the molecular drivers of this malignancy.

The molecular pathogenesis of UCS is a key area for exploration (16). Recent studies have suggested that UCS might share molecular characteristics with other aggressive endometrial cancers, such as mismatch repair deficiency, alterations in the PI3K/AKT pathway, and TP53muatations (1). These alterations may provide opportunities for more tailored therapeutic approaches.

In this case, immunohistochemical analysis demonstrated aberrant p53 expression (100%+), indicating a high-frequency TP53 mutation. As a key tumor suppressor, TP53 regulates cell cycle checkpoints, DNA repair, and apoptosis; loss of function due to mutation promotes uncontrolled proliferation, impaired apoptosis, and aggressive histopathological features—such as necrosis and extensive tumor thrombi—that portend a poor prognosis (1, 17). Immunohistochemistry also showed intact MMR proteins (MLH1, PMS2, MSH2, MSH6), consistent with a pMMR (mismatch repaired-proficient) status. The MMR system corrects errors during DNA replication; MMR deficiency (dMMR) drives genomic instability and increased tumor mutational burden, and in endometrial cancers, dMMR status correlates with responsiveness to immunotherapy due to enhanced neoantigen presentation (17).While pMMR tumors typically show limited response to PD-1 monotherapy, approximately 60% of UCS express PD-L1, supporting exploration of combinatorial immunotherapies such as pembrolizumab plus Lenvatinib. The PI3K/AKT pathway, which regulates tumor cell proliferation, survival, and metabolism, is frequently aberrantly activated in UCS (1, 18).Although PI3K/AKT pathway status was not assessed in this case, inhibitors targeting this pathway are under clinical evaluation and represent a potential therapeutic option.

Understanding how such molecular alterations intersect with benign pathologies like Brenner tumors could deepen insights into tumor heterogeneity. The current presence of both malignant and benign components in this patient may reflect a complex tumor microenvironment that influences tumor progression and treatment response (19).The bilateral ovarian Brenner tumors in this case particularly noteworthy, as they highlight a potentially underexplored interaction between benign and malignant processes. Brenner tumors are generally indolent with favorable prognoses, and their coexistence with UCS raises questions about shared molecular or environmental predisposing factors. Speculation includes the possibility that benign ovarian Brenner tumors act as precursor lesions or exert protective effects in the context of malignant uterine disease, or that a permissive tumor microenvironment facilitates the development of both tumor types through shared and complementary developmental pathways. This unprecedented association underscores the need to explore tumor heterogeneity and host-tumor interactions in UCS. Future studies profiling both UCS and concurrent Brenner tumors at the molecular level may clarify whether shared genetic, epigenetic, or microenvironmental factors underpin this duality, potentially informing therapeutic strategies for high-risk UCS subsets.

## 4 Conclusion

This case of uterine carcinosarcoma with concurrent bilateral ovarian Brenner tumors underscores the complexity of diagnosing and treating gynecological malignancies. It highlights the importance of comprehensive histological evaluation and the challenges associated with managing rare, biphasic tumors with poor prognostic outcomes.

## Data Availability

The original contributions presented in the study are included in the article/supplementary material. Further inquiries can be directed to the corresponding author.

## References

[1] Bogani G Ray-Coquard I Concin N Ngoi N Morice P Caruso G. Endometrial carcinosarcoma. Int J Gynecol. Cancer. (2023) 33(2):147–74. doi: 10.1136/ijgc-2022-004073, PMID: 36585027

[2] Siegel RL Giaquinto AN Jemal A. Cancer statistics, 2024. CA. Cancer J Clin. (2024) 74(1):12–49. doi: 10.3322/caac.21820, PMID: 38230766

[3] Yamada SD Burger RA Brewster WR Anton D Kohler MF Monk BJ. Pathologic variables and adjuvant therapy as predictors of recurrence and survival for patients with surgically evaluated carcinosarcoma of the uterus. Cancer. (2000) 88(12):2782–6. doi: 10.1002/1097-0142(20000615)88:12<2782::AID-CNCR17>3.0.CO;2-K, PMID: 10870061

[4] Kuhn E Ayhan A Shih I-M Seidman JD Kurman RJ. The pathogenesis of atypical proliferative Brenner tumor: an immunohistochemical and molecular genetic analysis. Mod Pathol Off J U. S. Can Acad Pathol Inc. (2014) 27(2):231–7. doi: 10.1038/modpathol.2013.142, PMID: 23887305 PMC4612641

[5] Lou Z Mei L Wan Z Zhang W Gao J. A report of twenty cases of ovarian Brenner tumor and literature review: a case series study. BMC Womens Health. (2024) 24(1):471. doi: 10.1186/s12905-024-03316-4, PMID: 39192213 PMC11348730

[6] Toboni MD Crane EK Brown J Shushkevich A Chiang S Slomovitz BM. Uterine carcinosarcomas: From pathology to practice. Gynecol. Oncol. (2021) 162 (1):235–41. doi: 10.1016/j.ygyno.2021.05.003, PMID: 34030871

[7] Matsuzaki S Klar M Matsuzaki S Roman LD Sood AK Matsuo K. Uterine carcinosarcoma: Contemporary clinical summary, molecular updates, and future research opportunity. Gynecol. Oncol. (2021) 160(2):586–601. doi: 10.1016/j.ygyno.2020.10.043, PMID: 33183764

[8] Huvila J Pors J Thompson EF Gilks CB. Endometrial carcinoma: molecular subtypes, precursors and the role of pathology in early diagnosis. J Pathol. (2021) 253(4):355–65. doi: 10.1002/path.5608, PMID: 33368243

[9] Maiorano MFP Cormio G Maiorano BA Loizzi V. Uterine carcinosarcoma (UCS): A literature review and survival analysis from a retrospective cohort study. Cancers. (2024) 16(23):3905. doi: 10.3390/cancers16233905, PMID: 39682097 PMC11640543

[10] Abu-Rustum N Yashar C Arend R Barber E Bradley K Brooks R. Uterine neoplasms, version 1.2023, NCCN clinical practice guidelines in oncology. J Natl Compr Canc. Netw. (2023) 21(2):181–209. doi: 10.6004/jnccn.2023.0006, PMID: 36791750

[11] Koh W-J Abu-Rustum NR Bean S Bradley K Campos SM Cho KR. Uterine neoplasms, version 1.2018, NCCN clinical practice guidelines in oncology. J Natl Compr Canc. Netw. (2018) 16(2):170–99. doi: 10.6004/jnccn.2018.0006, PMID: 29439178

[12] Wright JD Seshan VE Shah M Schiff PB Burke WM Cohen CJ. The role of radiation in improving survival for early-stage carcinosarcoma and leiomyosarcoma. Am J Obstet. Gynecol. (2008) 199(5):536.e1–8. doi: 10.1016/j.ajog.2008.04.019, PMID: 18511017

[13] Powell MA Filiaci VL Hensley ML Huang HQ Moore KN Tewari KS. Randomized phase III trial of paclitaxel and carboplatin versus paclitaxel and ifosfamide in patients with carcinosarcoma of the uterus or ovary: an NRG oncology trial. J Clin Oncol. (2022) 40(9):968–77. doi: 10.1200/JCO.21.02050, PMID: 35007153 PMC8937015

[14] Rauh-Hain JA Starbuck KD Meyer LA Clemmer J Schorge JO Lu KH. Patterns of care, predictors and outcomes of chemotherapy for uterine carcinosarcoma: A National Cancer Database analysis. Gynecol. Oncol. (2015) 139(1):84–9. doi: 10.1016/j.ygyno.2015.08.014, PMID: 26307402

[15] Giannini A Golia D'Augè T Bogani G Laganà AS Chiantera V Vizza E. Uterine sarcomas: A critical review of the literature. Eur J Obstet. Gynecol. Reprod Biol. (2023) 287:166–70. doi: 10.1016/j.ejogrb.2023.06.016, PMID: 37348383

[16] McConechy MK Hoang LN Chui MH Senz J Yang W Rozenberg N. In-depth molecular profiling of the biphasic components of uterine carcinosarcomas. J Pathol Clin Res. (2015) 1(3):173–85. doi: 10.1002/cjp2.18, PMID: 27499902 PMC4939881

[17] Bell DW Ellenson LH. Molecular genetics of endometrial carcinoma. Annu Rev Pathol. (2018) 14:339–67. doi: 10.1146/annurev-pathol-020117-043609, PMID: 30332563

[18] Bogani G Ray-Coquard I Concin N Ngoi NYL Morice P Enomoto T. Uterine serous carcinoma. Gynecol. Oncol. (2021) 162:226–34. doi: 10.1016/j.ygyno.2021.04.029, PMID: 33934848 PMC9445918

[19] Cherniack A Shen H Walter V Stewart C Murray BA Bowlby R. Integrated molecular characterization of uterine carcinosarcoma. Cancer Cell. (2017) 31(3):411–23. doi: 10.1016/j.ccell.2017.02.010, PMID: 28292439 PMC5599133

